# Oral exploration and food selectivity: A case-control study conducted in a multidisciplinary outpatient setting

**DOI:** 10.3389/fped.2023.1115787

**Published:** 2023-02-15

**Authors:** Marc Bellaïche, Véronique Leblanc, Jérôme Viala, Camille Jung

**Affiliations:** ^1^ Department of Pediatric Gastroenterology, Robert Debré Hospital, AP-HP, Paris, France; ^2^ Department of Clinical Research, Centre Hospitalier Intercommunal de Créteil, Créteil, France

**Keywords:** pediatric feeding disorders, sensory hypersensitivity, functional gastrointestinal disorders (FGID), toddlers, multidisciplinary setting

## Abstract

**Background:**

Pediatric feeding disorders (PFDs) are common, and their great phenotypic variability reflects the breadth of the associated nosological profiles. PFDs should be assessed and managed by multidisciplinary teams. Our study aimed to describe clinical signs of feeding difficulties in a group of PFD patients assessed by such a team, and to compare them with children in a control group.

**Methods:**

In this case-control study, case group patients 1 to 6 years old were consecutively recruited through the multidisciplinary unit for the treatment of pediatric feeding difficulties based at Robert Debré Teaching Hospital in Paris, France. Children with an encephalopathy, severe neurometabolic disorder, or genetic syndrome (suspected or confirmed) were excluded. Members of the control group, consisting of children with no feeding difficulties (i.e., Montreal Children's Hospital Feeding Scale scores below 60) or severe chronic diseases, were recruited from a day care center and 2 kindergartens. Data from medical histories and clinical examination related to mealtime practices, oral motor skills, neurodevelopment, sensory processing, and any functional gastrointestinal disorders (FGIDs) were recorded and compared between groups.

**Results:**

In all, 244 PFD cases were compared with 109 controls (mean ages: cases, 3.42 [±1.47]; controls, 3.32 [±1.17]; *P* = 0.55). Use of distractions during meals was much more among PFD children (cases, 77.46%; controls, 5.5%; *P* < 0.001), as was conflict during meals. While the groups did not differ in their members’ hand-mouth coordination or ability to grab objects, cases began exploring their environments later; mouthing, especially, was less common in the case group (cases, *n* = 80 [32.92%]; controls, *n* = 102 [94.44%]; *P* < 0.001). FGIDs and signs of visual, olfactory, tactile, and oral hypersensitivity were significantly more frequent among cases.

**Conclusion:**

Initial clinical assessments showed that, in the children with PFDs, normal stages of environmental exploration were altered, and that this was often associated with signs of sensory hypersensitivity and digestive discomfort.

## Introduction

Many parents consider their children picky eaters (1). In one cohort study that included 4,018 children, the prevalence of picky eating was 26% at 1.5 years of age, 28% at age 3, and 13% at age 6 (2). Variation in reported rates of prevalence can reflect the definitions applied, and whether the underlying data were obtained through questionnaires or examinations by specialists. Hence, Kovacic et al. observed that, among children age 5 or under, the prevalence of feeding disorders leading to appointments with specialists was much lower: between 2.1% and 3.5% per year (3).

The clinical picture of PFDs likewise varies greatly, from children who are light eaters to those with severe food selectivity. As shown by many studies, PFDs often emerge when infants reach stages requiring new skills, such as at the transition to complementary feeding or the switch from blended food to meals with pieces of food (4). Since 2013, the DSM-5 has recognized the existence of Avoidant/Restrictive Food Intake Disorder (ARFID), which is characterized by persistent failure to meet appropriate nutritional and energy requirements. Yet the DSM-5 provides a psychiatric definition, based on specific personality profiles and particularly focused on the nutritional consequences of feeding difficulties. Thus, it excludes young children who shun pieces of food but have a nutritionally balanced diet. In 2019, Goday et al. proposed a broader definition of PFD as “impaired oral intake that is not age-appropriate, and is associated with medical, nutritional, feeding skill, and/or psychosocial dysfunction” (5). In any case, unlike patients with anorexia nervosa, PFD patients do not present with body image disturbance.

PFDs are complex disorders driven by several factors—medical, developmental, nutritional, and psychosocial in nature. They are more frequent among children with a history of severe respiratory illnesses, digestive diseases, or neurodevelopmental disorders such as autism (6). In addition, delayed oral engagement and skill acquisition may cause feeding difficulties that expose children to the risk of malnutrition. Sensory sensitivity, marked by difficulty with the visual, olfactory, or tactile perception of foods, has been identified as another determinant of PFDs. In a large Japanese cohort of 3,728 children between 4 and 7 years old, the prevalence of ARFID was 1.3%. Among the ARFID children, according to their parents, 63% disliked “to eat food with a specific smell, taste, appearance, temperature, or a certain consistency/texture (e.g., crispy or soft)” (7).

Parents of PFD children often consider it a struggle to fulfill their parental role as feeders (8), and parental expectations and behaviors with regards to feeding vary between families. Kerzner et al. identify four caregiver feeding styles: (i) responsive—where parents share responsibility for feeding, establish a setting for meals, and positively respond to signs of hunger or satiety from their children; (ii) controlling—where parents ignore those signs, using force or rewards to get them to eat; (iii) indulgent—where parents feed their children whenever food is requested, preparing several dishes without enforcing limits; and (iv) neglectful—where parents ignore the nutritional and emotional needs of their children and do not interact with them during meals (9). With the aim of guiding management of PFD by specialists, Kerzner et al., in addition to identifying the profiles above, define three distinct eating behaviors by which to categorize these children: limited appetite, selective intake (i.e., accepted food is limited in texture, color, or variety), and fear of feeding (due to traumatic experiences).

PFDs thus span diverse etiologies and diagnoses. Though there have been many reviews of the literature, there is a lack of data on the clinical and developmental characteristics of young children receiving medical attention for PFDs. Murray et al. performed a retrospective analysis of a cohort of 129 pediatric patients referred for neurogastroenterology examinations in connection to functional gastrointestinal (GI) disorders (FGIDs). ARFID symptoms were more common in patients being treated for abdominal pain and lower GI symptoms (10). A Chinese study of 924 children ages 1 to 3—with or without feeding problems, according to the Montreal Children's Hospital Feeding Scale (MCHFS)—confirms the greater prevalence of FGIDs and poorer fine motor, personal, and social skills among children with feeding difficulties (11). However, while most authors recommend multidisciplinary treatment for PFDs (12), literature on the initial diagnostic assessment of these disorders by specialized teams within the outpatient setting is scarce.

The purpose of our study was to describe clinical signs linked with sensorimotor development and associated FGIDs in children between 1 and 6 years of age with or without PFDs.

## Patients and methods

### Patients

The patients in this case-control study were consecutively recruited through the multidisciplinary PFD unit at Robert Debré Teaching Hospital in Paris, France, between January 2017 and February 2021. The unit includes a pediatric gastroenterologist specialized in nutrition and a psychologist specialized in feeding difficulties among young children. At the end of an appointment, these professionals described their patient's feeding difficulties using a classification system based on the work of Kerzner et al. (9). Children with an encephalopathy, severe neurometabolic disorder, or genetic syndrome (whether suspected or confirmed) were excluded.

Members of the control group, also between 1 and 6 years old, were recruited at a day care center and 2 kindergartens in France. Criteria for control group inclusion were MCHFS scores (13) under 60 and the absence of genetic or severe neurological disorders.

The legal representatives of case and control children were informed of the study, which was approved by the CPP Sud-Est VI institutional review board (21.00685.000004) and registered with ClinicalTrials.gov (NCT05157633).

### Questionnaires

Standardized semistructured questionnaires were completed during appointments by examining specialists (for cases) or parents (for controls). Data collected through the case questionnaire included the reason for the appointment, medical or surgical history, a precise description of the feeding difficulty and mealtime practices, aspects of psychomotor development (i.e., general and fine motor function), and elements indicative of olfactory, visual, tactile, or oral sensory sensitivity.

The questionnaire for controls was limited to a subset of the above items, addressing the children's medical history (including any FGIDs) and development, as well as the typical course or structure of their meals (see questionnaire, Supplementary Data).

### Statistical analyses

Qualitative variables were described with numbers and percentages of patients concerned; and quantitative variables, with means (standard deviation) or medians (interquartile range), depending on their respective distributions. The groups were compared using Student's *t*-tests or Mann-Whitney tests for quantitative variables, and *χ*^2^ or Fisher's exact tests for qualitative variables. *P* values were considered significant if <0.05. Statistical analyses were performed using Stata/SE 16 (StataCorp, Texas, USA).

## Results

**Figure 1:**
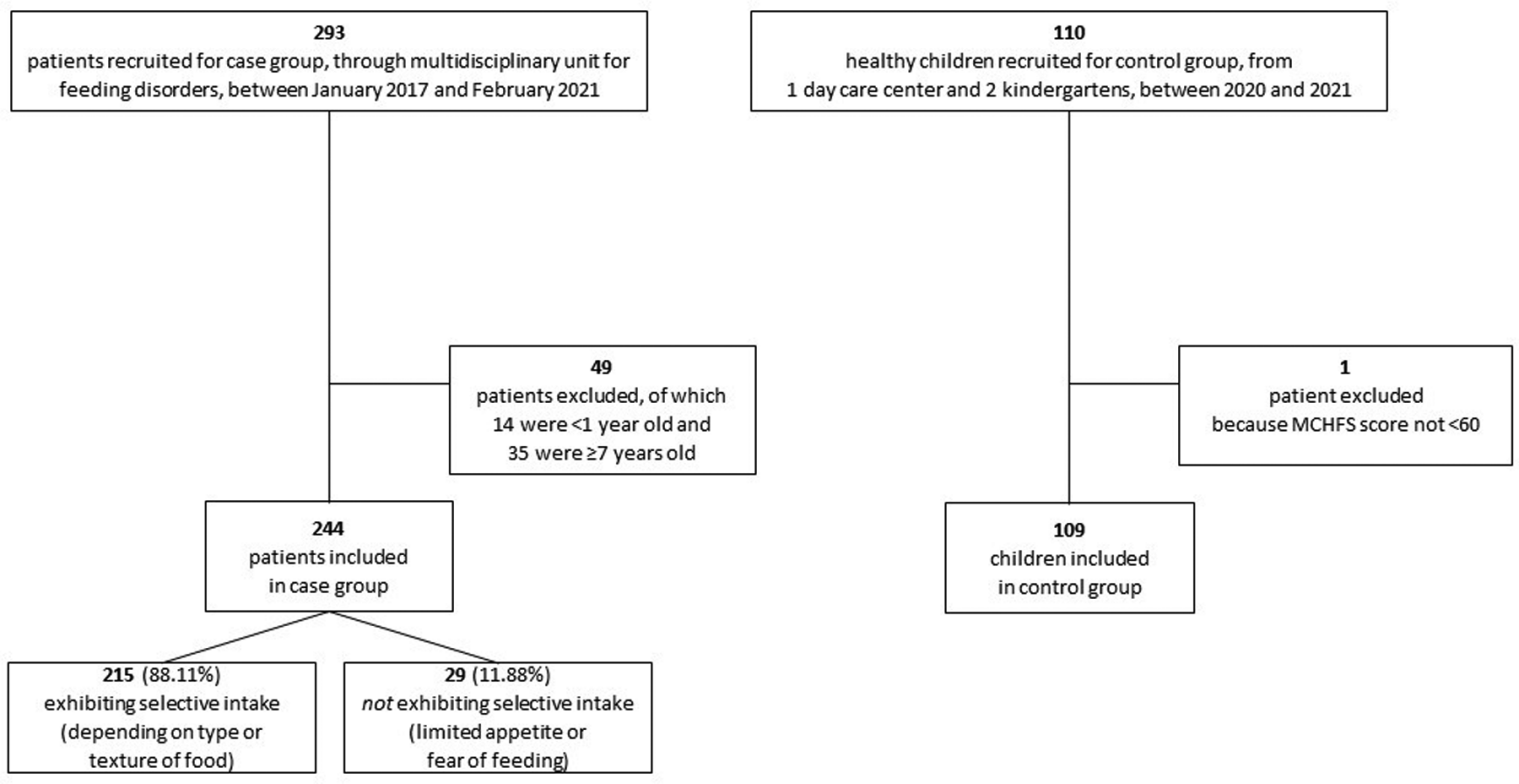
Study recruitment flowchart. MCHFS, Montreal Children's hospital feeding scale.

Between 2017 and 2021, 293 patients were examined at the Robert Debré multidisciplinary PFD unit. Of these, 244 met inclusion criteria. Besides these PFD patients, 109 children without feeding issues were recruited for the control group (Figure 1). The mean age for all study participants was 3.31 (± 1.38) years, and group means were similar (cases, 3.42 [± 1.47] years; controls, 3.32 [± 1.17] years; *P* = 0.55).

In the case group, the most common reason for an appointment indicated by parents was their children's selectivity in terms of categories (*n* = 213; 87.65%) or textures (*n* = 110; 45.27%) of foods consumed, with patients often exhibiting both kinds of selectivity (Table 1). At the end of an appointment, the pediatric specialist and psychologist classified the feeding disorder. Children exhibiting selective intake (*n* = 214; 87.70%) or sensory aversion for certain food categories made up the great majority of the case group (*n* = 169; 69.26%) (Table 1). Approximately 16% of PFD children had a “fear of feeding” and ∼8% had limited appetites.

**Table 1:** Reasons for appointments, classification of feeding disorders after multidisciplinary examination, and associated diagnoses.

Characteristics of patients (*N* = 244)	*n* (%)
**Reason for appointment**	
Selective intake	213 (87.29)
Refusal of pieces of food	110 (45.08)
Limited appetite	12 (4.92)
Difficult weaning, or enteral nutrition	7 (2.86)
Other feeding difficulties	12 (4.92)
**Medical classification**	
Selective intake	214 (87.70)
Sensory food aversion	169 (69.55)
Fear of feeding	38 (15.64)
Limited appetite	20 (8.20)
Difficult weaning, or enteral nutrition	2 (0.82)
**Associated diagnosis**	
Neurodevelopmental disorder/autism	39 (15.98)
Interaction problems	27 (11.06)

Most children ate at the table and with their parents, though this was more common in the control group (Table 2). Unlike controls, most case children had to be distracted by their parents during meals (cases, 77.46%; controls, 5.5%; *P* < 0.001). Additionally, meals were a source of conflict for more than half of case group families.

**Table 2:** Mealtime practices, oral motor skills, and psychomotor development. Data represents number (%) of patients with stated characteristics. Where there are missing data, the number (%) of children concerned is indicated in italics and brackets.

Characteristics	Total (*N* = 353)	Cases (*N* = 244)	Controls (*N* = 109)	*P* value
**Mealtime practices and child's behavior at table**				
Child eats at table	269 (89.94)	197 (82.08) [*2 (0.81*]	106 (99.07) [*1 (0.91)*]	<0.001
Family eats together at mealtimes	275 (87.03)	204 (84.30) [*2 (0.81)*]	105 (97.22) [*1 (0.91)*]	0.001
Child refuses food by turning head aside	113 (35.87)	109 (45.42) [*4 (1.63)*]	4 (3.67)	<0.001*
Parents must distract child during meals	195 (61.13)	189 (77.46)	6 (5.50)	<0.001
Child participates in meal preparation	177 (51.60)	86 (36.60) [*9 (3.68)*]	91 (84.26) [*1 (0.91)*]	<0.001
Parent-child conflict at mealtime	136 (42.63)	128 (52.46)	8 (7.34)	<0.001
Child keeps food in mouth	129 (40.82)	124 (51.45) [*3 (1.23)*]	5 (4.59)	<0.001*
Child eats selectively from meal presented	222 (70.48)	213 (88.38) [*3 (1.23)*]	9 (8.33) [*1 (0.91)*]	<0.001
**Oral motor skills evaluated by professionals during appointment**				
Proper mouth closure	329 (93.47)	220 (90.53) [*1 (0.41)*]	109 (100)	0.001
Good biting and chewing ability	228 (72.15)	153 (63.49) [*3 (1.23)*]	109 (100)	<0.001
Food chewed and swallowed	264 (75.43)	155 (64.32) [*3 (1.23)*]	109 (100)	<0.001
Tongue mobility	276 (78.86)	167 (69.29) [*3 (1.23)*]	109 (100)	<0.001
**Psychomotor development**				
First walked when >18 months old, where applicable (*n* = 320)	54 (16.88)	54 (24.77)	0	<0.001
Crawled	174 (50.43)	76 (31.93) [*6 (2.46)*]	98 (91.59) [*11 (10.09)*]	<0.001*
Psychomotor agitation (reported by parents or observed during multidisciplinary examination)	38 (11.91)	37 (15.16)	1 (0.92)	<0.001*
Postural problem (axial hypo- or hypertonia) observed during examination	23 (6.53)	21 (8.64) [*1 (0.41)*]	2 (1.83)	0.02*
Language delay (<20 words at age 2), where applicable (*n* = 289)	70 (24.22)	64 (32.65)	6 (6.45)	<0.001
Able to transfer contents between containers	301 (88.01)	192 (82.40) [*11 (4.50)*]	109 (100)	<0.001*
Able to stack three blocks when 12 months old	293 (86.69)	185 (80.79) [*15 (6.15)*]	108 (99.08)	<0.001*
Good hand-mouth coordination	310 (97.18)	235 (96.31)	109 (100)	0.06*
Object exploration through mouthing	182 (51.85)	80 (32.92)	102 (94.44) [*1 (0.91)*]	<0.001

* Fisher's exact test.

Although all controls had good oral motor skills—namely, proper mouth closure, tongue movement, and biting and chewing—the prevalence of deficits in these skills varied from 9.5% to 36.5% among case group children (Table 2).

Both groups were similar in terms of hand-mouth coordination and the ability to grab objects, but environmental exploration emerged later among cases. These children more frequently started walking when >18 months old (cases, *n* = 54 [24.77%]; controls, *n* = 0 [0%]; *P* < 0.001), did not crawl (∼69% of cases), and did not engage in exploratory mouthing of objects (cases, *n* = 80 [32.65%]; controls, *n* = 102 [94.44%]; *P* < 0.01). Language delays (<20 words by age 2) were likewise more frequently reported among children over 2 years of age in the case group: (cases, *n* = 64 [32.65%]; controls, *n* = 6 [6.45%]; *P* < 0.001).

Signs of visual, olfactory, tactile, or perioral/intraoral hypersensitivity were also sought (Table 3). They were significantly more prevalent in cases than among controls, who seldom exhibited any. For example, 18.83% (*n* = 45) of cases felt nauseous at the sight of food, and 32.22% (*n* = 77) did upon smelling food. Over half of the case group exhibited tactile hypersensitivity when walking on grass or sand or having lotion applied to the face. Children's urge to immediately clean their hands when smudged with paint was more common in the case group (cases, *n* = 124 [54.15%]; controls, *n* = 24 [22.02%]; *P* = 0.001), while playing with food on their plate with their hands was more frequent in the control group (cases, *n* = 57 [23.95%]; controls, *n* = 92 [85.19%]; *P* < 0.001). Signs of peri- and intraoral hypersensitivity—particularly when pieces of food were offered—were also overwhelmingly present among cases.

**Table 3:** *Signs suggestive of sensory hypersensitivity.* Data represents number (%) of patients with stated characteristics. Where there are missing data, the number (%) of children concerned is indicated in italics and brackets.

Signs of hypersensitivity	Total (*N* = 353)	Cases (*N* = 244)	Controls (*N* = 109)	*P* value
**Visual**				
Child feels nauseous upon sight of food	47 (14.97)	45 (18.83)	2 (1.83)	<0.001
**Olfactory**				
Feels nauseous upon smelling food	80 (25.56)	77 (32.22)	3 (2.75)	<0.001
**Tactile**				
Enjoys taking bath	312 (88.39)	204 (83.61)	108 (99.08)	<0.001*
Enjoys having lotion applied to body	278 (79.43)	179 (73.97) [*2 (0.81)*]	99 (91.67) [*1 (0.91)*]	0.001
Enjoys having lotion applied to face	210 (60.00)	120 (49.59) [*2 (0.81)*]	90 (83.33) [*1 (0.91)*]	<0.001
Enjoys walking on grass	197 (58.81)	100 (43.86) [*16 (6.55)*]	97 (90.65) [*2 (1.83)*]	<0.001
Enjoys walking on sand	198 (59.64)	99 (44) [*19 (7.78)*]	99 (92.52) [*2 (1.83)*]	<0.001
Quickly cleans hands when gets paint on them	148 (48.68)	124 (54.15) [*15 (6.14)*]	24 (22.02)	0.001
Quickly cleans hands when gets food on them	192 (60.95)	173 (72.08) [*4 (1.63)*]	19 (17.43)	<0.001
Constantly cleans hands	19 (5.96)	19 (7.79)	0 (0)	0.001*
Refuses to touch food	167 (52.85)	161 (66.80) [*3 (1.23)*]	6 (5.50)	<0.001
Plays, or once played, with food (using spoon or hands)	232 (67.05)	134 (56.30) [*6 (2.46)*]	99 (91.67)	<0.001
Plays, or once played, with food using hands	149 (43.06)	57 (23.95) [*6 (2.46)*]	92 (85.19)	<0.001
**Peri- and intraoral**				
Feels nauseous when offered food with smooth texture	68 (21.66)	68 (28.33) [*4 (1.63)*]	0 (0) [*1 (0.91)*]	<0.001*
Feels nauseous when offered pieces of food	168 (53.67)	166 (69.46) [*4 (1.63)*]	2 (1.85) [*1 (0.91)*]	<0.001*
Feels nauseous when food in mouth	72 (22.57)	70 (28.96) [*4 (1.63)*]	2 (1.83)	<0.001*
Cries when food in mouth	59 (18.50)	56 (22.95)	3 (2.75)	<0.001*
Tolerates toothbrushing	259 (77.08)	156 (68.42) [*16 (6.55)*]	103 (95.37) [*1 (0.91)*]	<0.001*
Cries when balm applied to lips	110 (34.48)	108 (44.26)	2 (1.83)	<0.001*

* Fisher's exact test.

Finally, the prevalence of FGIDs (e.g., constipation, gastroesophageal reflux, or a history of infant colic) was significantly higher in the case group (Table 4).

**Table 4:** *Prevalence of functional gastrointestinal disorders.* Data represents number (%) of patients with stated characteristics. GI, gastrointestinal.

GI disorder	Total (*N* = 353)	Cases (*N* = 244)	Controls (*N* = 109)	*P* value
Functional GI disorder	175 (49.72)	134 (54.92)	41 (37.96) [*1 (0.91)*]	0.003
Constipation	137 (38.81)	117 (47.95)	20 (18.35)	<0.001
History of infant colic	31 (8.78)	12 (4.92)	19 (17.43)	<0.001
Gastroesophageal reflux	87 (24.65)	69 (28.28)	18 (16.51)	0.018
History of food allergy	15 (4.72)	15 (6.17) [*1 (0.41)*]	0	0.007*

* Fisher's exact test.

Results are similar if we exclude from our study population autistic children and those with neurodevelopmental disorders (Supplementary Data).

## Discussion

This study considered a large group of children with feeding disorders examined as outpatients by a specialized multidisciplinary team following referral by primary care physicians (pediatricians or general practitioners). Delayed neuromotor skill acquisition (affecting walking, language, and environmental exploration) and signs of sensory sensitivity were much more frequent among these patients than in the control group of children without feeding disorders.

The great prevalence of sensory hypersensitivity observed in this study has also been reported by other authors. Dinkler et al. submitted questionnaires to parents of children diagnosed with ARFID per DSM-5 criteria. The most commonly reported driver of food avoidance was “sensory sensitivity to food characteristics” (7). For their large Norwegian cohort, Steinsbekk et al. showed that sensory hypersensitivity (as demonstrated by a score that integrated reactions to tactile, visual, oral, gustatory, olfactory, and auditory stimuli) in 4-year-old children predicted picky eating at age 6 (14). In our group of PFD children, we observed a stepped increase in the prevalence of signs of hypersensitivity affecting the various senses: nearly a fifth exhibited visual hypersensitivity to foods; a third, olfactory; over half, tactile; and over two-thirds, intraoral.

Another notable phenotypic trait more common among the cases was the presence of FGIDs. This association has been highlighted by other authors, especially for abnormal bowel movements (i.e., diarrhea or constipation) (10, 11). It is possible that low fiber intake contributes to constipation in PFD children. However, Tappin et al. showed that increasing fiber in children's diet does not suffice to treat constipation (15). It is therefore likely that digestive discomfort contributes to these children's feeding difficulties.

The clinical profiles of PFD children varies. Some have identifiable underlying diseases, which may be organic (e.g., celiac disease, encephalopathy, dysphagia, or organ insufficiency) or psychiatric (e.g., depression, infantile anorexia, or parental expectations not suited to children's eating behaviors). Others have feeding difficulties that might be described as functional, with or without sensory hypersensitivity. The PFD patients in our study, recruited through a multidisciplinary unit specialized in feeding disorders to which they had been referred by their primary physicians, may be considered to fall into this latter category. This possibility was acknowledged when Rome IV criteria were drawn up in 2016. In their review of Rome IV criteria for neonates and toddlers, Benninga et al. indicate (seventh recommendation for future research) that, in addition to those described in their article, other disorders may need to be recognized as FGIDs in this age group—“particularly those related to feeding disorders” (16). Indeed, we suggest that the PFD presented by cases in our study, in the absence of any underlying organic or psychiatric disorder, be designated *functional toddler feeding disorder* (FTFD). FTFD may thus be considered an FGID that presents alone or alongside other FGIDs (17).

Its foremost characteristic is difficulty managing sensory stimuli from foods associated with delayed acquisition of neuromotor skills, although there is uncertainty as to whether this developmental delay is the cause or effect of the disorder. Is the neuromotor delay—e.g., late (or no) crawling or late walking—the cause of impeded environmental exploration, or does the child's primary hypersensitivity hamper object exploration and the normal stages of neurodevelopment?

To properly eat, infants require eating skills and a suitable environment. Specifically, the following conditions have to be met: (i) the preparation and serving of the meal, as well as (ii) mealtime practices and the general environment, must be adapted; and the child must (iii) possess the required skills, (iv) have the ability to explore the environment, objects, and the food presented, and (v) tolerate the associated sensory stimuli. Ramos et al. demonstrated that 94% of the 70 children in their study who had feeding difficulties did not possess the necessary feeding skills. These skills are normally acquired when children are between 6 and 24 months old, and they include the ability to handle different food textures, eat with their hands, drink from a cup and with a straw, and assume a proper position for eating (18).

**Figure 2:**
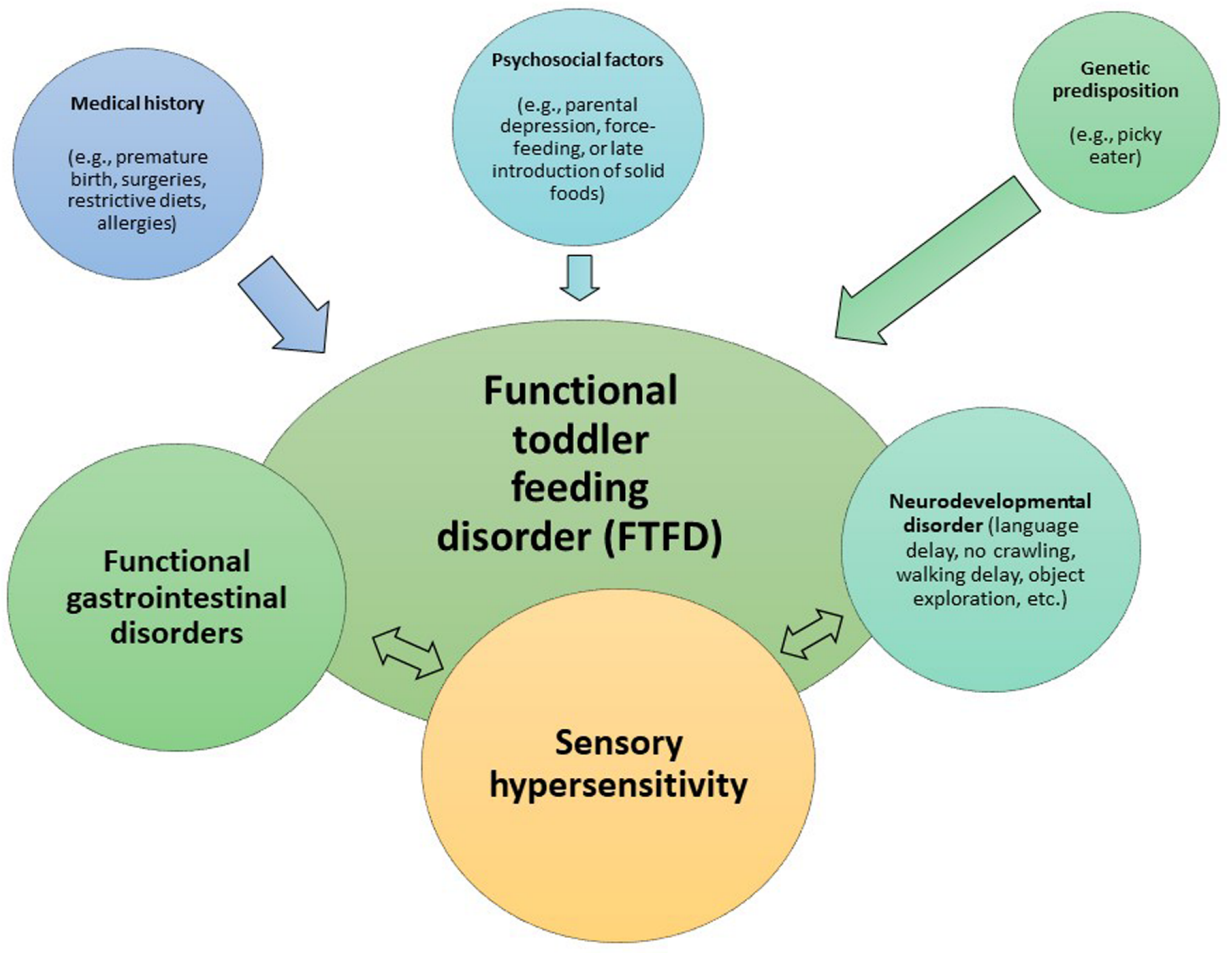
Biopsychosocial model of functional toddler feeding disorder.

Feeding difficulties may arise if any of these skills acquired in normal development is lacking. Leblanc, a coauthor of this article and clinical psychologist specialized in feeding difficulties, has written—from the perspective of a child with such a difficulty—that “my mouth isn’t touching what my eyes, hands, and nose haven’t mastered” (19). As is the case for the visceral hypersensitivity that may explain certain FGIDs (20), and in accordance with the biopsychosocial model, sensory hypersensitivity may be driven by multiple factors, including genetic predisposition (e.g., children are light eaters), psychosocial aspects (e.g., demanding family environment characterized by force-feeding, parents with psychiatric illnesses, or delayed introduction of solid foods), and medical history (e.g., premature birth, nasogastric intubation, prescription of restrictive diet, or repeated surgery) (Figure 2). Moreover, children with autism spectrum disorder (ASD) are no exception. Just as FGIDs are more common among ASD children than in the general population (21), so is FTFD (22). It has long been known, through administration of the Dunn sensory profile, that sensory sensitivity is prevalent in ASD children (23).

## Conclusion

Two observations may be made on the basis of our initial evaluation of toddlers with PFDs: Firstly, these patients do not exhibit the usual progression through the developmental stages of environmental exploration—especially *via* mouthing of objects, which normally emerges between the ages of 8 and 10 months old. Secondly, there is a greater prevalence of functional disorders associated with these PFDs, which we propose calling FTFD.

## Data Availability

The datasets presented in this article are not readily available because the legal representatives have not authorized the sharing of the data. Requests to access the datasets should be directed to Camille Jung, camille.jung@chicreteil.fr.
