# Supplementary material for: Oral exploration and food selectivity: A case-control study conducted in a multidisciplinary outpatient setting
Source: Front Pediatr. 2023 Feb 15;11:1115787. doi: 10.3389/fped.2023.1115787 (PMC9977162; doi:10.3389/fped.2023.1115787)
Supplement: Supplementary file 1 [file Table1.docx]

**Supplementary data**

**Table S1*. Mealtime practices, oral motor skills, and psychomotor development in patients with feeding disorders (excluding those with neurodevelopmental disorders) and controls.*** Data represents number (%) of patients with stated characteristics. Where there are missing data, the number (%) of children concerned is indicated in italics and brackets.

| **Characteristics** | **Total**  **(*N* = 314)** | **Cases**  **(*N* = 205)** | **Controls**  **(*N* = 109)** | ***P* value** |
| --- | --- | --- | --- | --- |
| **Mealtime practices and child’s behavior at table** | | | | |
| Child eats at table | 272 (88.31) | 166 (82.59) [*1 (0.48)*] | 106 (99.07) [*1 (0.91)*] | <0.001 |
| Family eats together at mealtimes | 279 (89.71) | 174 (85.71) [*1 (0.48)*] | 105 (97.22) [*1 (0.91)*] | 0.001 |
| Child refuses food by turning head aside | 113 (35.87) | 109 (45.42) [*4 (1.95)*] | 4 (3.67) | <0.001* |
| Parents must distract child during meals | 166 (52.87) | 160 (78.05) | 6 (5.50) | <0.001 |
| Child participates in meal preparation | 167 (54.93) | 76 (38.78) [*9 (4.39)*] | 91 (84.26) [*1 (0.91)*] | <0.001 |
| Parent-child conflict at mealtime | 122 (38.85) | 114 (55.61) | 8 (7.34) | <0.001 |
| Child keeps food in mouth | 106 (34.08) | 101 (50.0) [*3 (1.46)*] | 5 (4.59) | <0.001* |
| Child eats selectively from meal presented | 187 (87.68) | 178 (87.68) [*1 (0.48)*] | 9 (8.33) [*1 (0.91)*] | <0.001 |
| **Oral motor skills evaluated by professionals during appointment** | | | | |
| Proper mouth closure | 305 (92.77) | 196 (95.61) | 109 (100) | 0.026 |
| Good biting and chewing ability | 256 (82.32) | 147 (72.77) [*3 (1.46)*] | 109 (100) | <0.001 |
| Food chewed and swallowed | 258 (82.69) | 149 (73.40) [*2 (0.98)*] | 109 (100) | <0.001 |
| Tongue mobility | 266 (85.26) | 157 (77.34) [*2 (0.98)*] | 109 (100) | <0.001 |
| **Psychomotor development** |  |  |  |  |
| First walked when >18 months old, where applicable (*N* = 285) | 31 (10.88) | 31 (16.94) | 0 | <0.001 |
| Crawled | 163 (53.27) | 65 (32.66) [*6 (2.92)*] | 98 (91.59) [*11 (10.09)*] | <0.001* |
| Psychomotor agitation (reported by parents or observed during multidisciplinary examination) | 21 (6.69) | 20 (9.76) | 1 (0.92) | 0.002* |
| Postural problem (axial hypo- or hypertonia) observed during examination | 16 (5.11) | 14 (6.86) [*1 (0.48)*] | 2 (1.83) | 0.06* |
| Language delay (<20 words at age 2), where applicable (*N* = 259) | 50 (19.31) | 44 (26.51) | 6 (6.45) | <0.001 |
| Able to transfer contents between containers | 278 (91.45) | 169 (86.67) [*10 (4.80)*] | 109 (100) | <0.001* |
| Able to stack three blocks when 12 months old | 272 (90.67) | 164 (85.86) [*14 (6.82)*] | 108 (99.08) | <0.001* |
| Good hand-mouth coordination | 309 (98.41) | 200 (97.56) | 109 (100) | 0.16* |
| Object exploration through mouthing | 167 (53.53) | 65 (31.86) | 102 (94.44) [*1 (0.91)*] | <0.001 |

******* Fisher’s exact test

**Table S2. *Signs suggestive of sensory hypersensitivity in patients with feeding disorders (excluding those with neurodevelopmental disorders) and controls.*** Data represents number (%) of patients with stated characteristics. Where there are missing data, the number (%) of children concerned is indicated in italics and brackets.

| **Signs of hypersensitivity** | **Total (*N* = 314)** | **Cases**  **(*N* = 205)** | **Controls**  **(*N* = 109)** | ***P* value** |  |
| --- | --- | --- | --- | --- | --- |
| **Visual** | | | | | |
| Child feels nauseous upon sight of food | 40 (12.74) | 38 (18.54) | 2 (1.83) | <0.001 |  |
| **Olfactory** | | | | | |
| Feels nauseous upon smelling food | 70 (22.29) | 67 (32.68) | 3 (2.75) | <0.001 |  |
| **Tactile** | | | | | |
| Enjoys taking bath | 289 (92.04) | 181 (88.29) | 108 (99.08) | <0.001* |  |
| Enjoys having lotion applied to body | 258 (82.96) | 159 (73.97) [*2 (0.98)*] | 99 (91.67) [*1 (0.91)*] | 0.003 |  |
| Enjoys having lotion applied to face | 199 (63.99) | 109 (53.69) [*2 (0.98)*] | 90 (83.33) [*1 (0.91)*] | <0.001 |  |
| Enjoys walking on grass | 186 (62.63) | 89 (46.84) [*15 (7.31)*] | 97 (90.65) [*2 (1.83)*] | <0.001 |  |
| Enjoys walking on sand | 189 (64.07) | 90 (47.87) [*17 (8.29)*] | 99 (92.52) [*2 (1.83)*] | <0.001 |  |
| Quickly cleans hands when gets paint on them | 129 (42.72) | 105 (54.40) [*12 (5.85)*] | 24 (22.02) | <0.001 |  |
| Quickly cleans hands when gets food on them | 168 (53.85) | 149 (73.40) [*2 (0.98)*] | 19 (17.43) | <0.001 |  |
| Constantly cleans hands | 19 (6.07) | 19 (9.25) | 0 (0) | 0.001* |  |
| Refuses to touch food | 143 (45.83) | 137 (67.49) [*2 (0.98)*] | 6 (5.50) | <0.001 |  |
| Plays, or once played, with food (using spoon or hands) | 214 (69.48) | 116 (58.00) [*5 (2.43)*] | 99 (91.67) | <0.001 |  |
| Plays, or once played, with food using hands | 139 (45.13) | 47 (23.50) [*5 (2.43)*] | 92 (85.19) | <0.001 |  |
| **Peri- and intraoral** | | | | | |
| Feels nauseous when offered food with smooth texture | 53 (17.15) | 53 (26.37) [*4 (1.95)*] | 0 (0) [*1 (0.91)*] | <0.001* |  |
| Feels nauseous when offered pieces of food | 140 (45.16) | 138 (68.32) [*3 (1.46)*] | 2 (1.85) [*1 (0.91)*] | <0.001* |  |
| Feels nauseous when food in mouth | 60 (19.11) | 58 (28.90) | 2 (1.83) | <0.001* |  |
| Cries when food in mouth | 46 (14.65) | 43 (20.98) | 3 (2.75) | <0.001* |  |
| Tolerates toothbrushing | 239 (79.93) | 136 (71.20) [*14 (6.82)*] | 103 (95.37) [*1 (0.91)*] | <0.001* |  |
| Cries when balm applied to lips | 90 (28.66) | 88 (42.93) | 2 (1.83) | <0.001* |  |

******* Fisher’s exact test

**Table S3*: Prevalence of functional gastrointestinal disorders in patients with feeding eating disorders (excluding those with neurodevelopmental disorders) and controls.*** Data represents number (%) of patients with stated characteristics. GI = gastrointestinal.

| **GI disorder** | **Total (*N* = 314)** | **Cases (*N* = 205)** | **Controls (*N* = 109)** | ***P* value** |
| --- | --- | --- | --- | --- |
| Functional GI disorder | 150 (47.92) | 109 (47.92) | 41 (37.96) [*1 (0.91)*] | 0.01 |
| Constipation | 118 (37.58) | 98 (47.80) | 20 (18.35) | <0.001 |
| History of infant colic | 31 (9.87) | 12 (5.85) | 19 (17.43) | <0.001 |
| Gastroesophageal reflux | 73 (23.25) | 55 (26.83) | 18 (16.51) | 0.039 |
| History of food allergy | 12 (3.83) | 12 (5.88) [*1 (0.48)*] | 0 | 0.01* |

******* Fisher’s exact test
