# Supplementary material for: Oral exploration and food selectivity: A case-control study conducted in a multidisciplinary outpatient setting
Source: Front Pediatr. 2023 Feb 15;11:1115787. doi: 10.3389/fped.2023.1115787 (PMC9977162; doi:10.3389/fped.2023.1115787)
Supplement: Supplementary file 2 [file Table2.docx]

**Questionnaire: Your Child’s Eating Habits**

| Initials of first and last names: | Age: |
| --- | --- |

| **MEDICAL HISTORY** |  |
| --- | --- |
| Has your child suffered from previous illnesses?  If so, please describe briefly: | Yes No |
| Has your child been previously hospitalized?  If so, please describe briefly: | Yes No |
| **PSYCHOMOTOR DEVELOPMENT** |  |
| Was your child able to grab objects using the thumb and index finger at about 9 months old? | Yes No |
| At about what age did your child acquire hand-mouth coordination (i.e., ability to grab and mouth an object)? | ___ months |
| Did your child ever crawl or move by scooting on his/her buttocks? | Yes No |
| Has your child had problems acquiring language skills? | Yes No |
| Did your child start walking at a late age (i.e., after 18 months)? | Yes No |
| Did your child often use a pacifier? | Yes No |
| **HISTORY OF FIGD** |  |
| Does your child have a history of . . .  gastroesophageal reflux, i.e., regurgitation more than 3 times a day for 3 weeks?  constipation requiring treatment?  infant colic, noted in his/her health record? | Yes No  Yes No  Yes No |

**MEALS**

| **Textures** | Does your child . . .  cry or turn his/her head aside at the sight of a baby bottle or spoon?  feel nauseous when offered food with smooth texture?  exhibit food selectivity (“picky eating”), only liking certain kinds of food?  refuse to eat pieces of food (i.e., not blended)?  keep pieces of food in his/her mouth, rather than swallow them?  feel nauseous when offered pieces of food? | Yes No  Yes No  Yes No  Yes No  Yes No  Yes No |
| --- | --- | --- |
| **Mealtime Interaction and Pleasure** | Does your child . . .  eat meals with the rest of the family?  help prepare meals with the rest of the family?  agree to come to the table for a meal?  enjoy coming to the table for a meal?  enjoy tasting food? | Yes No  Yes No  Yes No  Yes No  Yes No |

**SENSORY SENSITIVITY**

| Does your child . . .  enjoy taking a bath?  enjoy having lotion applied to his/her body?  enjoy having lotion applied to his/her face? | Yes No  Yes No  Yes No |
| --- | --- |
| enjoy walking barefoot on grass?  enjoy walking barefoot on sand? | Yes No  Yes No |
| engage in exploratory mouthing of objects? | Yes No |
| cry when someone comes near to his/her face?  feel nauseous when someone touches his/her mouth? | Yes No  Yes No |
| cry when someone touches his/her mouth? | Yes No |
| brush his/her teeth easily? | Yes No |
| feel nauseous upon smelling certain foods? | Yes No |
| feel the need to clean his/her hands after touching modeling clay or paint? | Yes No |
| feel the need to clean his/her hands after touching certain foods? | Yes No |
| feel nauseous at the sight of certain foods? | Yes No |
| refuse to touch certain foods? | Yes No |
| feel nauseous upon touching certain foods? | Yes No |
| like to play with food?  If so,  with a spoon or other utensil?  with his/her hands? | Yes No  Yes No  Yes No |
